# Onlinepsychotherapie in Zeiten der Corona-Pandemie: Querschnittsbefragung deutscher Psychotherapeuten

**DOI:** 10.1007/s00278-021-00519-0

**Published:** 2021-07-05

**Authors:** Franziska Marie Lea Beck-Hiestermann, Denise Kästner, Antje Gumz

**Affiliations:** Fachbereich Psychosomatik und Psychotherapie, Psychologische Hochschule Berlin, Am Köllnischen Park 2, 10179 Berlin, Deutschland

**Keywords:** Pandemien, Telekommunikation, Face-to-face-Therapie, Persönliche Zufriedenheit, Technologieakzeptanz, Pandemics, Telecommunications, Face-to-face therapy, Personal satisfaction, Technology acceptance

## Abstract

**Theoretischer Hintergrund:**

In Reaktion auf die durch die „coronavirus disease 2019“ (COVID-19) verursachte Pandemie konnte Psychotherapie im Einzelsetting in Deutschland unbegrenzt online durchgeführt werden. Haltungen und Erfahrungen von Psychotherapeuten (PT) bezüglich Onlinetherapie (OT) wurden jedoch allgemein und besonders mit Blick auf die Pandemiesituation bislang wenig untersucht.

**Ziel der Arbeit:**

Ziele der Studie waren, 1) die Nutzungshäufigkeit von OT während des ersten Lockdowns, 2) die Zufriedenheit mit OT vs. „Face-to-face“-Therapie sowie 3) die Technologieakzeptanz und -erfahrung insgesamt und in Abhängigkeit vom Richtlinienverfahren zu untersuchen.

**Material und Methode:**

Deutsche PT (approbiert und in Ausbildung; verhaltenstherapeutisch [VT, 45,6 %], tiefenpsychologisch [TP, 34,5 %], analytisch [AP, 14 %], systemisch [SYS, 5,8 %]) wurden mithilfe einer Onlineerhebung zu demografischen und therapeutischen Daten, durchgeführter OT, Zufriedenheit mit OT vs. Face-to-face-Therapie (Zufriedenheitsfragebogens für Therapeuten, ZUF-THERA) und Technologieakzeptanz (Unified Theory of Acceptance and Use of Technology 2 Questionnaire, UTAUT) befragt.

**Ergebnisse:**

Die 174 teilnehmenden Therapeuten (Alter M = 44,73 Jahre, SD ± 12,79; 81,6 % Frauen) gaben an, dass der durchschnittliche Anteil von OT an der gesamten therapeutischen Tätigkeit während des Lockdowns 43,09 % betrug, wobei sich signifikante Unterschiede zwischen den Richtlinienverfahren zeigten (TP, VT > AP). Die Zufriedenheit mit OT erwies sich als signifikant niedriger als mit Face-to-face-Therapien und unterschied sich zwischen den Verfahren nicht. Vorerfahrungen mit OT hatten insgesamt 23,6 % der Therapeuten und vermehrt systemisch arbeitende im Vergleich zu VT- oder AP-Therapeuten. Verhaltenstherapeuten gaben häufiger an, Spaß an der OT zu haben, als TP- und APler. Auch nahmen sie einen stärkeren sozialen Einfluss (beispielsweise durch Kollegen) bei der Nutzung von OT wahr als die TPler.

**Schlussfolgerung:**

Die Nutzungshäufigkeit von OT nahm während des ersten Lockdowns (März bis Mai 2020) sprunghaft zu (43 %, zum Vergleich das frühere Abrechnungslimit der Krankenkassen: 20 %). Die Zufriedenheit mit der OT war prinzipiell hoch, jedoch signifikant niedriger als mit Face-to-face-Therapien. Weiterführende Untersuchungen, die die Gründe im Detail analysieren, werden dringend angeraten.

## Abstract (en)

**Theoretical background:**

As a reaction to the coronavirus diseases 2019 (COVID-19) pandemic, in individual settings psychotherapy could be conducted online to an unlimited extent in Germany. The attitudes and experiences of psychotherapists with respect to online therapy (OT) have so far been generally poorly studied and particularly with a view to the situation during the pandemic.

**Objective:**

The aim of the study was to examine 1) the frequency of utilization of OT during the first lockdown, 2) the satisfaction with OT versus face-to-face therapy and 3) the technology acceptance experience overall and with respect to the guideline procedures.

**Material and methods:**

German psychotherapists licensed and in training, cognitive-behavioral (CB 45.6%), analytic (AP 14%), depth-psychological (DP 34.5%), systemic (SYS 5.8%), were invited to participate in an online survey on demographic and therapeutic data, use of OT, satisfaction with OT vs. face-to-face therapy (*Zufriedenheitsfragebogen für Therapeuten*, ZUF-THERA) and technology acceptance (Unified Theory of Acceptance and Use of Technology 2 Questionnaire, UTAUT).

**Results:**

The 174 participating psychotherapists (mean age = 44.73 years, SD ± 12.79; female 81.6%) reported that the average proportion of OT in the total therapeutic activity during the lockdown was 43.09%, with significant differences between guideline procedures (DP, CB > AP). The satisfaction with OT proved to be significantly lower than with face-to-face therapy and did not differ between the procedures. Prior experience with OT was reported by 23.6% of therapists overall and was higher among those working systemically compared to CB or AP therapists. Therapists working in CB experienced more enjoyment with OT than those working in DP and AP as well as perceived a stronger social influence (e.g. through colleagues) in the use of OT than therapists working in DP.

**Conclusion:**

The frequency of use of OT soared during the first lockdown (March–May 2020, 43% in comparison to the former limit covered by health insurances of 20%). In principle, therapists were highly satisfied with OT but significantly lower than with face-to-face therapy. Further studies analyzing the reasons for this in detail are urgently recommended.

Im Rahmen der Coronapandemie erfolgten in Deutschland drastische Beschränkungen des gesamten öffentlichen Lebens und die schnelle Implementierung des Kontaktverbots. Im Zuge dessen nahm die Bedeutung und Nutzung der psychotherapeutischen Onlinetherapie (OT) innerhalb kürzester Zeit massiv zu. Viele Therapeuten begannen mehr oder weniger gezwungenermaßen mit OT, ohne vorab Zeit für Schulungen oder weitere Unterstützung zu haben. Welche Haltung zu und Erfahrungen mit der OT sich daraus für Therapeuten unterschiedlicher Schulen ergaben, wurde in der vorliegenden Studie untersucht.

## Einleitung

Im Dezember 2019 traten in Wuhan in China erstmals Fälle mit Pneumonien unbekannter Ursache auf; in der Folge konnte rasch ein neues Coronavirus als Ursache der inzwischen als „coronavirus disease 2019“ (COVID-19) bezeichneten Erkrankung identifiziert werden. Seither sind Infektionen in vielen Ländern weltweit bestätigt worden. Als Reaktionen auf den Verlauf der Pandemie beschlossen die Länder unterschiedliche, restriktive Maßnahmen, die das öffentliche Leben stark einschränkten. Zur weiteren Eingrenzung der Pandemie einigten sich am 22.03.2020 Bund und Länder auf ein umfassendes Kontaktverbot (Besprechung der Bundeskanzlerin mit den Regierungschefinnen und Regierungschefs der Länder 2020). Eine solch drastische Beschränkung des gesamten öffentlichen Lebens war in dieser Form erstmalig in Deutschland. Dies hatte und hat anhaltend auch Implikationen für die psychotherapeutische Versorgung. Richtlinienpsychotherapie, psychotherapeutische Sprechstunde und probatorische Gespräche können während der Zeit der COVID-19-Pandemie unbegrenzt online durchgeführt werden, vorausgesetzt, dass diesen ein persönlicher Patientenkontakt zu Eingangsdiagnostik, Indikationsstellung und Aufklärung vorausgegangen ist (Bundespsychotherapeutenkammer 2020). Somit nahm die Bedeutung der Onlinetherapie (OT) innerhalb kürzester Zeit massiv zu, und es kam zum sprunghaften Anstieg von OT-Settings. Es ist anzunehmen, dass die Umstellung eine große Herausforderung für Therapeuten und Patienten bedeutete. Der Wechsel des Settings von „face-to-face“ zu OT könnte sich auf die therapeutische Beziehung auswirken (Eichenberg 2020). Eine gute therapeutische Allianz gilt als einer der wichtigsten Wirkfaktoren und als gut belegter Prädiktor für den psychotherapeutischen Behandlungserfolg (Horvath et al. 2011). Erste Forschungsergebnisse zeigen, dass eine stabile und positive therapeutische Beziehung auch im Onlinesetting implementiert werden kann (Eichenberg und Hübner im Druck). Die Einstellungen und Haltungen von Therapeuten gegenüber OT sind i. Allg. wenig untersucht (Klug et al. 2008). In einem Review analysierten Connolly et al. (2020) die Forschungsliteratur, die sich mit der Einstellung oder Zufriedenheit der Therapeuten gegenüber Online-Video-Therapie beschäftigte. Es zeigte sich, dass Behandler generell eine positive Einstellung gegenüber OT haben, obwohl mehrere Nachteile beschrieben werden. Da es jedoch keinen Goldstandard für die Messung der Konstrukte „Zufriedenheit“ und Einstellung gegenüber Videotherapie gibt, sind die Ergebnisse nur vorsichtig interpretier- und vergleichbar (Connolly et al. 2020).

Die meisten Studien, die die Erfahrungen der Behandler zu OT und Face-to-face-Therapie verglichen, kommen zu dem Ergebnis, dass die Studienteilnehmenden Letztere als wünschenswerter empfanden. So beschreiben psychodynamische Therapeuten OT als etwas weniger effektiv als Face-to-face-Sitzungen (Gordon et al. 2015). Des Weiteren wurden Face-to-face-Sitzungen von den Therapeuten signifikant höher bewertet, in Bezug auf die Zufriedenheit (Mayworm et al. 2019; Ruskin et al. 2004; Schopp et al. 2000), die Zielbildung, die Aufgabenerfüllung und die Entwicklung einer therapeutischen Beziehung (Ertelt et al. 2011). Die Bewertung der OT reichte von angemessen (Kopel et al. 2001), über gleichwertig (Elford et al. 2001) bis hin zur akzeptablen Alternative (Elford et al. 2000; Thomas et al. 2017). Trotz signifikanter Unterschiede in der Zufriedenheit mit OT und Face-to-face-Therapie war diese generell für beide Settings hoch (Mayworm et al. 2019; Ruskin et al. 2004; Ertelt et al. 2011).

Die dargestellten Studien bilden jedoch eine Besonderheit der COVID-19-Situation nicht ab: Durch das schnelle Implementieren der Restriktionen musste die Umstellung auf OT rasch erfolgen. Folglich begannen viele Therapeuten mehr oder weniger gezwungenermaßen mit OT, ohne vorab Zeit für Schulungen oder weitere Unterstützung zu haben. Eine auf OT während der COVID-19-Pandemie bezogene Studie (Aafjes-van Doorn et al. 2020) zeigte, dass technische Schwierigkeiten als größte Herausforderung bei der Umstellung auf OT genannt wurden. Dies bildete sich ebenfalls im Review von Connolly et al. (2020) ab, in dem eine positive Einstellung gegenüber Technik ein Prädiktor bezüglich der Zufriedenheit damit war. Des Weiteren verweist eine österreichische Studie darauf, dass OT nicht gleichermaßen von Therapeuten unterschiedlicher Verfahren angenommen wird und es Unterschiede beim Erleben des Wechsels von Face-to-face zu OT gibt. Psychotherapeuten humanistischer und psychodynamischer Verfahren erlebten den Wechsel zu OT i. Allg. als positiver als Verhaltenstherapeuten (Humer et al. 2020).

Vor diesem Hintergrund hatte die vorliegende Studie das Ziel, die Nutzung von und die Zufriedenheit mit OT während des ersten Lockdowns zu untersuchen. Konkret sollte betrachtet werden, wie hoch: 1) der Anteil der während des ersten Lockdowns durchgeführten OT, 2) die Zufriedenheit der Therapeuten mit OT im Vergleich zu Face-to-face-Therapie sowie 3) die Technologieakzeptanz der Therapeuten war. Dabei sollten alle Richtlinienverfahren im Vergleich untersucht werden.

## Methode

### Teilnehmer und Rekrutierung

Die Daten wurden mithilfe einer querschnittlichen, anonymen Onlineumfrage erhoben. Eingeschlossen werden konnten psychologische oder ärztliche Psychotherapeuten (approbiert oder in Ausbildung), die während des ersten COVID-19-bedingten Lockdowns von März bis Mai 2020 mindestens einmal eine Online-Video-Therapie angeboten haben. Dabei wurden Telefontherapie ebenso wie Coaching und Beratungsangebote ausgeschlossen.

Die Rekrutierung erfolgte durch Anschreiben von 265 psychotherapeutischen Ausbildungsinstituten mit Internetpräsenz und 5965 niedergelassenen Therapeuten, die ihre Praxis in gängigen Therapeutensuchmaschinen gelistet hatten. Weiterhin wurden soziale Medien, beispielsweise *Facebook* oder *Instagram*, zur Rekrutierung verwendet. Zusätzlich befand sich am Ende der Befragung ein Text zur Studieneinladung mit der Bitte um Weiterleitung an potenziell interessierte Kollegen.

Die Erhebung ersteckte sich vom 01.12.2020 bis zum 31.12.2020. Innerhalb dieses Zeitraums konnten die Daten von 184 Therapeuten erhoben und hiervon von 174 Therapeuten analysiert werden konnten.

### Messinstrumente

Die Erhebung beinhaltete demografische Daten, wie Alter, Geschlecht, Familienstand, aktuelle Partnerschaft, Wohnsituation, Region, Anzahl der Kinder, Anzahl der Geschwister, Migrationshintergrund, Bildungsabschluss und Beschäftigung. Weiterhin wurden Daten zu therapeutischer Tätigkeit und Ausbildung erfasst, wie Studienfach, Approbation, Verfahren, Weiterbildungen, (Einzel)Selbsterfahrungsstunden, Zufriedenheit mit Selbsterfahrung, eigene Therapieerfahrungen, Anzahl der aktuell und insgesamt behandelten Patienten jeweils im ambulanten und im (teil-) stationären Setting, Anzahl der Supervisionsstunden sowie Jahr der Approbation (bzw. Ausbildungsbeginn). Auch Online-Skills wie die Intensität der PC- und Internetnutzung ebenso wie Vorerfahrungen mit OT wurden ermittelt. Zudem wurde im Hinblick auf die Zeit während des ersten Lockdowns erfragt, wie viele Patienten behandelt wurden, die Anzahl der Patienten, die zur OT wechselten/diese ablehnten, sowie die Gründe für die Ablehnung.

#### Zufriedenheit.

Die Zufriedenheit mit der OT wurde mithilfe des Zufriedenheitsfragebogens für Therapeuten (ZUF-THERA; Puschner et al. 2005) gemessen. Der ZUF-THERA ist eine Adaptation des ZUF‑8, der ein Instrument zur Erfassung der globalen Patientenzufriedenheit, beispielsweise nach einem Klinikaufenthalt, darstellt. (Item-Beispiele: „Wie würden Sie die Qualität der Behandlung, welche Sie erhalten haben, beurteilen?“ oder „Haben Sie die Art von Behandlung erhalten, die Sie wollten?“) Der ZUF-THERA untersucht auf Basis des ZUF‑8 die Zufriedenheit der Therapeuten. Anders als der ZUF‑8 besteht der ZUF-THERA aus 6 Items, nämlich den Items 1, 2, 3, 5, 6 und 8 des ZUF‑8. Diese Items wurden auf den Therapeuten umformuliert und bezogen sich konkret auf den Lockdown von März bis Mai 2020. (Item-Beispiele: „Wie würden Sie die Qualität Ihrer Onlinetherapie beurteilen?“ oder „In welchem Ausmaß hat die Onlinetherapie den Bedürfnissen der Patienten entsprochen?“) Es gibt auch hier 4 Antwortmöglichkeiten, ohne eine neutrale Position. Die Punktwerte der einzelnen Items werden zu einem Gesamt-Score zusammengefasst, der von 6 bis 24 Punkten reicht und die globale Zufriedenheit der Therapeuten widerspiegelt. In der Validierungsstudie von Puscher zeigte der ZUF-THERA ein Cronbachs α von 0,82, was einer guten internen Konsistenz entspricht (Blanz 2015). Für die vorliegende Studie wurde der Fragebogen 2‑mal ausgefüllt: einmal bezogen auf die Face-to-face-Therapie, einmal hinsichtlich der OT.

#### Technologieakzeptanz.

Diese wurde mithilfe des Unified Theory of Acceptance and Use of Technology 2 Questionnaire (UTAUT; Venkatesh et al. 2003) untersucht, der ein etabliertes Instrument zur Messung dieses Konstrukts darstellt. Die verwendete validierte deutsche Übersetzung (Harborth und Pape 2018) umfasst 8 Subskalen, bestehend aus jeweils 3 bis 4 Items. Sie werden mithilfe einer 7‑stufigen Likert-Skala mit den Endpunkten „stimme überhaupt nicht zu“ und „stimme absolut zu“ gemessen. Die interne Konsistenz der Subskalen beträgt zwischen Cronbachs α 0,733 und 0,951 (akzeptabel bis hoch; Blanz 2015).

Ergänzend wurden Freitextfelder verwendet, die im Rahmen einer separaten qualitativen Untersuchung ausgewertet wurden (Gumz et al. im Druck). Die qualitative Untersuchung basiert auf insgesamt 1392 schriftlichen Einzelaussagen, die inhaltsanalytisch ausgewertet und zu 88 Sub- und 9 Oberkategorien zusammengefasst wurden.

### Analysen und Statistik

Von 184 rekrutierten Probanden mussten 10 ausgeschlossen werden. Gründe für den Ausschluss waren fehlende berufliche Qualifikation (beispielsweise Heilpraktiker) oder unvollständig ausgefüllten Daten (>5 %). Mit den Daten der 174 Therapeuten umfassenden Stichprobe wurden deskriptive Analysen zu Stichprobencharakterisierung, Nutzung von PC und Internet i. Allg. und von OT während des Lockdowns berechnet. Zusätzlich wurden „analyses of variance“ (ANOVA) durchgeführt, um Unterschiede zwischen den Richtlinienverfahren bezüglich Technologieakzeptanz sowie Nutzung und Zufriedenheit von OT und Face-to-face-Therapie zu untersuchen. Die Voraussetzungen zur Durchführung der ANOVA wurden vorab jeweils geprüft. In 3 Fällen (UTAUT-Dimension Aufwandserwartung, prozentualer Anteil an OT und Vorerfahrung mit OT) war die Voraussetzung der Varianzhomogenität nicht erfüllt. In der Folge wurde ein Post-hoc-Test (nach Games-Howell), der die Varianzungleichheit toleriert, genutzt.

Bei der Analyse fehlender Werte zeigte sich, dass die Variablen zu den zentralen Fragestellungen (Nutzungshäufigkeit, Vorerfahrung und Zufriedenheit mit OT, Technologieakzeptanz, Nutzung von PC und Internet, Richtlinienverfahren) nahezu vollständig ausgefüllt wurden. Einzelne fehlenden Werte (*n* = 1 bis 8 Teilnehmer) waren in wenigen Variablen zu verzeichnen. Vor diesem Hintergrund wurde sich gegen Imputationsverfahren entschieden, und die Ergebnisse wurden auf Basis des Originaldatensatzes mit Verweis auf fehlende Angaben bzw. eine reduzierte Stichprobengröße berichtet.

Alle statistischen Analysen erfolgten mithilfe von IBM SPSS Statistics 27.

## Ergebnisse

### Stichprobenbeschreibung

Die Mehrheit der untersuchten Therapeuten war weiblich (*n* = 142, 81,6 %), und das Durchschnittsalter betrug 44,73 Jahre (Standardabweichung [SD] ± 12,79); Altersunterteilung nach Richtlinienverfahren: Tab. 1. Der überwiegende Anteil der Therapeuten hatte Psychologie studiert (79,3 %), war bereits approbiert (63,2 %) und besaß die Behandlungserlaubnis für Erwachsene (79,3 %).

**Table 1:** 

Merkmale	Stichprobe (*n* = 174)	
	**M**	**SD**
*Alter (Jahre)*	44,73	± 12,79
Verhaltenstherapeuten	44,48	± 11,84
Tiefenpsychologisch arbeitende Therapeuten	41,78	± 10,56
Analytisch arbeitenden Psychotherapeuten	52,71	± 12,32
Systemisch arbeitende Therapeuten	55,20	± 8,04
	***n***	**%**
*Geschlecht*		
Männlich	30	17,4
Weiblich	142	81,6
Divers	0	0
Keine Angabe	2	1,2
*Familienstand*		
Ledig	59	33,9
Verheiratet	91	52,3
Getrennt/geschieden	18	10,3
Sonstiges	6	3,5
*Partnerschaft*		
Ja	141	81,0
Nein	33	19,0
*Studium*		
Psychologie	138	79,3
Medizin	15	8,6
Sonstiges	21	12,1
*Approbation*		
Ja	110	63,2
Nein	64	36,8
*Richtlinienverfahren*		
VT	78	45,6
TP	59	34,5
AP	24	14,0
SYS	10	5,8
Ohne Angabe	3	1,7
*Kinder- und Jugendlichen-PT*		
Ausschließich Kinder und Jugendliche	7	4,1
Ausschließlich Erwachsene	134	79,3
Beides	28	16,1
Keine Angabe	5	2,9

*VT* Verhaltenstherapie, *TP* Tiefenpsychologie, *AP* analytische Psychotherapie, *SYS* systemische Psychotherapie, *PT* Psychotherapie, *M* Mittelwert, *SD* Standardabweichung

Die Richtlinienverfahren bildeten sich wie folgt ab: Verhaltenstherapie (VT) mit 45,6 %, tiefenpsychologisch fundierte Psychotherapie (TP) mit 34,5 %, analytische Psychotherapie (AP) mit 14 % und systemische Psychotherapie (SYS) mit 5,8 %; keine Angabe machten 2,9 % der Teilnehmenden (*n* = 5).

Bezüglich der Berufserfahrung behandelten die untersuchten Therapeuten durchschnittlich 16 Patienten aktuell im ambulanten Setting und 317 im Verlauf ihrer bisherigen beruflichen Karriere. Stationär wurde durchschnittlich ein Patient aktuell und wurden 119 im Verlauf des Lebens behandelt.

Für weitere Ergebnisse: Tab. 1 und 2.

**Table 2:** 

Versorgungsform	Patienten	
	M (± SD)	Range
*Aktuell*		
Ambulant	16 (± 9,26)	0–44
Stationär	1,2 (± 3,48)	0–21
*Lifetime*		
Ambulant	317,10 (± 606,67)	3–4000
Stationär	119,49 (± 183,38)	0–1200

*M* Mittelwert, *SD* Standardabweichung

### Nutzung von OT während des ersten Lockdowns

Die Therapeuten haben durchschnittlich mit der Hälfte ihrer Patienten mindestens eine therapeutische Sitzung online durchgeführt (52 %; Tab. 3). Der durchschnittliche Anteil von OT an der gesamten therapeutischen Tätigkeit betrug 43,09 % während der Dauer des Lockdowns. Dabei gab es jeweils eine verhältnismäßig große Gruppe, die OT eher selten einsetzte (45,4 %, OT-Anteil 0–25 %) und die OT sehr viel nutzte (35,9 %, OT-Anteil 75–100 %). Eine mittlere Nutzung war tendenziell eher selten (15,5 % und 13,2 % für OT Anteile 25–50 % resp. 50–75 %). Etwas mehr als ein Fünftel der Patienten waren nicht bereit, zur OT zu wechseln (21,67 %), ein weiteres Drittel der Patienten wollte die Therapie online mithilfe reduzierter Stundenfrequenz fortsetzen, um beispielsweise lange Therapiepausen zu überbrücken (30,14 %).

**Table 3:** 

Fragestellung	Stichprobe	
	**Anteil (%)**	
Ausgehend von der Gesamtzahl Ihrer wöchentlichen Therapiesitzungen ...	**M (±** **SD)**	**Range**
Wie groß war der der durchschnittliche, prozentuale Anteil an Onlinetherapie während des „Lockdowns“?	43,09 (± 34,17)	0–100
Mit wie viel Prozent Ihrer Patienten haben Sie während des „Lockdowns“ mindestens eine Onlinetherapiesitzung durchgeführt?	52,61 (± 34,82)	0–100
Wie viel Prozent Ihrer Patienten waren bereit, mit reduzierter Stundenfrequenz zum Onlineformat zu wechseln (um beispielsweise lange Therapiepausen zu vermeiden)?	30,14 (± 37,34)	0–100
Wie viel Prozent Ihrer Patienten waren gar nicht bereit, zum Onlineformat zu wechseln?	21,67 (± 26,32)	0–99
	**Anzahl (*n*)**	
	**M (±** **SD)**	**Range**
Wie viele Patienten haben ihre laufenden Face-to-face-Therapietermine im Zusammenhang mit COVID-19 oder dem „Lockdown“ abgesagt (z. B. aus Angst vor Ansteckung, fehlender Kinderbetreuung …)?	4,90 (± 2,08)	0–6
Wie viele Stunden Onlinetherapie haben Sie während des „Lockdowns“ insgesamt circa durchgeführt? (Angabe in Stunden)	67,63 (± 74,55)	0–400

*M* Mittelwert, *SD* Standardabweichung

Die Nutzungshäufigkeit von OT unterschied sich signifikant bei Psychotherapeuten unterschiedlicher Verfahren (Tab. 4). Im Post-hoc-Test (nach Games-Howell) zeigte sich, dass tiefenpsychologisch (42 % OT) und verhaltenstherapeutisch arbeitende Psychotherapeuten (49 % OT) signifikant häufiger OT anwendeten als analytisch arbeitende Kollegen (21 % OT). Systemische Psychotherapeuten (47 % OT) unterschieden sich nicht signifikant von den TP-, AP- oder VT-Kollegen.

**Table 4:** 

–	Quadratsumme	df	Mittel der Quadrate	F	Signifikanz	Post hoc
*ZUF*						
Online	30,26	3	10,09	1,126	0,340	–
Face-to-face	3,37	3	1,12	0,424	0,736	–
Differenz	47,26	3	15,75	1,546	0,205	–
*Anteil (%) der OT während des Lockdowns*	14342,91	3	4780,97	4,363	0,005	TP > AP* VT > AP***

*ZUF* Fragebogen zur Messung der Therapeutenzufriedenheit, *OT* Onlinetherapie, *Post hoc* Signifikanz im Post-hoc-Test, *df* „degrees of freedom“, *F* F-Wert

*p < 0,05, **p < 0,01, ***p < 0,001

### Zufriedenheit mit OT im Vergleich zur Face-to-face-Therapie

**Figure 1:**
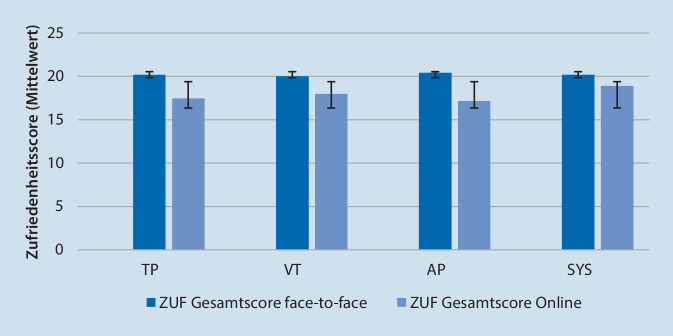

Durchschnittlich betrug der Zufriedenheitsscore verfahrensübergreifend 17,82 (SD ± 3,04) für die OT und 20,19 (SD ± 1,65) für die Face-to-face-Therapie, wobei höhere Werte mit größerer Zufriedenheit assoziiert sind (Abb. 1). Der Unterschied zwischen der Zufriedenheit mit OT und Face-to-face-Therapien ist signifikant (*t*(172) = 9,77, *p* < 0,001). Das heißt, dass Therapeuten im Schnitt zufriedener mit den Face-to-face-Therapien als mit den OT sind.

Bezüglich der Differenz der Zufriedenheitsscores von Face-to-face vs. OT konnten keine signifikanten Unterschiede zwischen den Richtlinienverfahren festgestellt werden (Tab. 4).

### Technologieakzeptanz und -erfahrung unter Psychotherapeuten

#### Nutzung von PC und Internet

**Figure 2:**
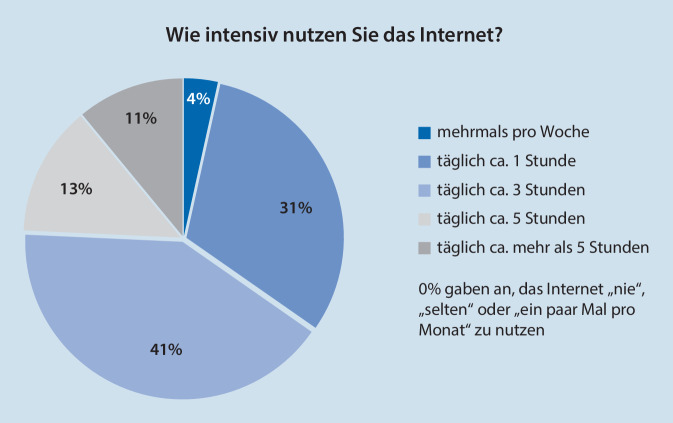

Alle Teilnehmer nutzten das Internet mindestens mehrmals pro Woche bis hin zu mehr als 5 h am Tag (Abb. 2). Kein Teilnehmer nutzte das Internet „nie“, „selten“ oder „ein paar Mal pro Monat“. Die Teilnehmer nutzen seit durchschnittlich 24,74 Jahren (SD ± 6,33 Jahre) einen PC, mit einem Range von 7 bis 40 Jahren (je nach Alter der Teilnehmer). Die täglich verbrachte Zeit am PC betrug durchschnittlich 4,11 h (SD ± 2,26 h; Range 1–12 h).

#### Vorerfahrung mit OT

**Figure 3:**
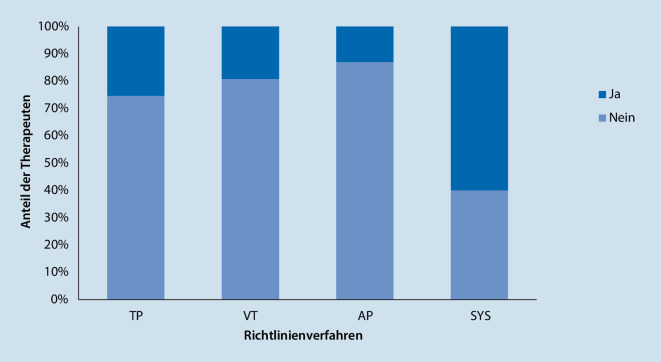

Es hatten 23,6 % (*n* = 41) der Therapeuten bereits Vorerfahrung mit OT, 75,9 % (*n* = 132) hatten keine Vorerfahrungen, und *n* = 1 (0,6 %) machte keine Angabe. Bezogen auf die Richtlinienverfahren sind unterschiedliche prozentuale Häufigkeiten zu beobachten. In der Gruppe der SYS hatten 60 % der Therapeuten bereits Vorerfahrungen mit OT, bei den VT waren es 19,2 %, bei den psychodynamischen Verfahren waren 25,4 % der TP und 13 % der AP vorerfahren (Abb. 3). Die Unterschiede zwischen den Richtlinienverfahren sind signifikant, (*F*(3, 166) = 3,406, *p* = 0,019). Dabei handelt es sich um einen mittleren Effekt von *ηp*^*2*^ = 0,058 (Cohen 1988). Post hoc zeigten sich Unterschiede zwischen den Richtlinienverfahren SYS vs. AP (*p* = 0,019) und SYS vs. VT (*p* = 0,023).

#### Technologieakzeptanz

Auf den 9 Dimensionen der Technologieakzeptanz (UTAUT) ergaben sich verfahrensübergreifend folgende Werte (theoretischer Skalenrange: Tab. 4): Angewohnheit M = 6,21 (SD ± 3,70), Leistungserwartung M = 7,09 (SD ± 3,06), Aufwandserwartung M = 7,64 (SD ± 2,99), sozialer Einfluss M = 5,40 (SD ± 3,15), hedonistische Motivation M = 2,66 (SD ± 1,77), Preis und Wert M = 4,40 (SD ± 1,37), erleichternde Bedingungen M = 14,56 (SD ± 2,55), Verhaltensabsicht 7,57 (SD ± 3,26) und Angst M = 6,87 (SD ± 4,96).

Im Vergleich der Richtlinienverfahren hinsichtlich der Dimensionen fanden sich auf den Subskalen hedonistische Motivation und sozialer Einfluss signifikante Unterschiede. Verhaltenstherapeuten hatten signifikant mehr Spaß an der Nutzung von OT (Beispielitem: „Onlinetherapie macht mir Spaß“) als analytisch und tiefenpsychologisch arbeitende Kollegen. Dies traf ebenso auf systematisch arbeitende Therapeuten im Vergleich zu analytischen Therapeuten zu. Bei der Dimension sozialer Einfluss (Beispielitem: „Personen, deren Meinung ich schätze, empfehlen mir, dass ich Onlinetherapie nutze“) konnten signifikante Unterschiede zwischen Verhaltenstherapeuten und Tiefenpsychologen gefunden werden; der soziale Einfluss stellte sich bei VT größer dar. Mit Blick auf alle weiteren Subskalen der UTAUT ergaben sich keine Unterschiede zwischen Therapeuten unterschiedlicher Orientierung. Das ausgeübte Verfahren hatte keine Auswirkungen darauf, ob die Therapeuten beabsichtigen, OT nach der Coronapandemie weiterzunutzen. Einen Überblick gibt Tab. 5.

**Table 5:** 

	Approbierte und Psychotherapeuten in Ausbildung									
	TP		VT		AP		SYS			
	M	± SD	M	± SD	M	± SD	M	± SD	Theoretischer Skalenrange	Post hoc
Angewohnheit	5,80	± 3,55	6,67	± 3,73	4,75	± 3,74	7,50	± 3,44	0–12	–
Leistungserwartung	6,76	± 3,18	7,45	± 2,84	5,83	± 3,14	8,70	± 2,95	0–12	–
Aufwandserwartung	7,36	± 2,67	8,09	± 2,84	6,33	± 3,62	8,20	± 3,46	0–12	–
Hedonistische Motivation	2,24	± 1,52	3,08	± 1,79	1,79	± 1,72	3,60	± 1,51	0–6	VT > TP*, VT > AP**, SYS > AP*
Preis und Wert	4,17	± 1,45	4,59	± 1,23	4,08	± 1,53	4,70	± 1,34	0–6	–
Sozialer Einfluss	4,68	± 3,11	6,15	± 2,94	4,38	± 3,47	5,50	± 2,80	0–12	VT > TP*
Erleichternde Bedingungen	14,24	± 2,58	14,82	± 2,51	14,63	± 2,32	14,00	± 2,94	0–18	–
Verhaltensabsicht	6,93	± 3,19	8,04	± 3,20	6,46	± 3,18	9,20	± 3,12	0–12	–
Angst	7,71	± 5,12	6,33	± 4,86	7,67	± 4,86	5,70	± 4,72	0–24	–

UTAUT-Dimensionen (mit Itembeispiel): Angewohnheit („Onlinetherapie zu nutzen, ist bei mir zur Angewohnheit geworden“), Leistungserwartung („Ich empfinde Onlinetherapie in meinem Alltag als nützlich“), Aufwandserwartung („Ich finde, Onlinetherapie ist einfach“), sozialer Einfluss („Personen, deren Meinung ich schätze, empfehlen mir, dass ich Onlinetherapie nutze“), hedonistische Motivation („Onlinetherapie zu nutzen, macht Spaß“), Preis und Wert („Zum derzeitigen Aufwand bietet Onlinetherapie einen guten Nutzen“), erleichternde Bedingungen („Ich habe die notwendigen Ressourcen zum Nutzen von Onlinetherapie“), Verhaltensabsicht („Ich beabsichtige, in der Zukunft auch weiterhin Onlinetherapie zu nutzen“), Angst („Ich habe Bedenken, Onlinetherapie zu benutzen“)

*M* Mittelwert, *SD* Standardabweichung, *TP* tiefenpsychologisch fundierte Psychotherapie, *VT* Verhaltenstherapie, *AP* analytische Psychotherapie, *SYS* systemische Psychotherapie

*Post hoc* Signifikanz im Post-hoc-Test (nach Bonferroni): *p < 0,05, **p < 0,01

## Diskussion

### Interpretation der Studienergebnisse

Die Studie hatte zum Ziel, die Nutzung von und Zufriedenheit mit OT im Vergleich zur Face-to-face-Therapie zu untersuchen. Obwohl die Therapeuten unterschiedliche Vorerfahrungen mit OT hatten, der Wechsel pandemiebedingt schnell vonstattengehen musste sowie qualitativ diverse Befürchtungen und Ängste berichtet wurden (Gumz et al. im Druck), haben sie mit durchschnittlich der Hälfte der Patienten mindestens eine OT-Sitzung durchgeführt. Dabei war die Zufriedenheit der Therapeuten mit Face-to-face-Therapien signifikant höher als mit OT, jedoch gab es keine signifikanten Unterschiede zwischen den Richtlinienverfahren. Im Hinblick auf die Technologieaffinität waren nur auf 2 der 9 Subskalen signifikante Unterschiede zu verzeichnen.

Dies lässt die Vermutung zu, dass evtl. Vorbehalte und Ablehnung bezüglich OT sich nicht bewahrheiten, wenn man „gezwungen“ ist, diese durchzuführen. Durch die COVID-19-bedingte Notwendigkeit der Umstellung auf OT musste sich der Großteil der Psychotherapeuten mit dem Medium auseinandersetzen. So behandeln die befragten Therapeuten im Schnitt aktuell 16 Patienten und gaben an, dass durchschnittlich 6 Patienten pandemiebedingt Termine absagten. In mehr als einem Drittel der laufenden Therapien gab es also coronabedingte Ausfälle.

Dass Psychotherapeuten bisher wenig praktische Erfahrungen damit gemacht haben, begründet sich nicht zuletzt auch durch die eingeschränkten Möglichkeiten zur Abrechnung mit den Kostenträgern (Haun et al. 2020). Vor der Pandemie galt grundsätzlich, dass in einem Quartal maximal 20 % der jeweiligen Leistung per Video erbracht werden dürfen (Bundespsychotherapeutenkammer 2019). Mit einem durchschnittlichen OT-Anteil von 43 % waren die Leistungen also deutlich über den zuvor erlaubten Höchstgrenzen.

Die Einstellungen gegenüber Technik, abgebildet mithilfe der UTAUT-Dimensionen, unterschieden sich bei den Richtlinienverfahren nur auf 2 Dimensionen signifikant. Dies kann zum einen daran liegen, dass Internet- und PC-Nutzung in der vorherrschenden technologisierten Gesellschaft zur alltäglichen Gewohnheit geworden sind, was sich in den Nutzungshäufigkeiten (Abb. 2) widerspiegelt. Zum anderen könnte es infolge des Formats der Onlineerhebung einen Selektionsbias bei der Stichprobenauswahl hin zu eher technikaffinen Studienteilnehmern gegeben haben.

Im Hinblick auf die UTAUT-Dimensionen mit signifikanten Unterschieden zwischen den Richtlinienverfahren hatten Verhaltenstherapeuten signifikant höhere Werte auf der Hedonismusdimension („OT macht Spaß“) als tiefenpsychologisch und analytisch arbeitende Kollegen. Dies steht im Einklang mit aktuellen Vorbefunden (Békés und Aafjes-van Doorn 2020).

Auch haben analytische Psychotherapeuten signifikant weniger OT angewendet als die anderen Richtlinienverfahren. Womöglich wird die OT von ihnen stärker als „notwendiges Übel“ in Zeiten der Pandemie gesehen. Wenn OT jedoch zur Anwendung kommt, ist die Zufriedenheit unter den OT eher wenig anwendenden analytischen Psychotherapeuten ebenso hoch wie unter den Therapeuten der anderen Richtlinienverfahren. Bezüglich des sozialen Einflusses (beispielsweise der Bedeutung des kollegialen Rates oder eines Vorbildes) zeigten sich signifikante Unterschiede zwischen Verhaltenstherapeuten und Tiefenpsychologen, was die Bedeutung von kollegialem Austausch für die Meinungsbildung gegenüber OT unterstreicht.

Therapeuten aller Verfahren waren im Mittel etwas unzufriedener mit der OT im Vergleich zur Face-to-face-Therapie. Die Gründe werden in einer qualitativen Studie (Gumz et al. im Druck) untersucht; erste Hinweise gibt eine „Blitzumfrage“ der Deutschen Psychotherapeutenvereinigung. Diese zeigte, dass die Wirksamkeit von OT von zwei Dritteln der Befragten als geringer eingeschätzt wird, was sich möglichweise auch auf die Zufriedenheit damit auswirkt (Deutsche Psychotherapeutenvereinigung 2020). Nichtsdestotrotz kann die OT über die Zeit der Coronapandemie hinaus eine Lösung für andere Versorgungsprobleme darstellen: z. B. Unterversorgung auf dem Land, Versorgung von mobilitätseingeschränkten Patienten, oder im Kinder- und Jugendbereich bei getrennten Elternteilen, die in großer räumlicher Distanz leben. Somit kann die Pandemie als Chance gesehen werden, OT nicht nur als „notwendiges Übel“, sondern in verschiedenen Aspekten auch als gute Ergänzung zur Face-to-face-Therapie zu betrachten.

### Stärken und Schwächen der Studie

In Anbetracht des sehr kurzen Rekrutierungszeitraums konnte eine relativ hohe Stichprobengröße mit einem breiten Altersspektrum und mit Ausnahme der systemischen Psychotherapeuten auch eine gute Gruppengröße in den Richtlinienverfahren erreicht werden. Zu den Einschränkungen zählen die geringe Anzahl der systemisch arbeitenden Therapeuten, die dazu führt, dass die Vergleiche mit dieser Gruppe als weniger zuverlässig betrachtet werden müssen, sowie die internetbasierte Erhebung, die einen Selektionsbias mit sich bringen könnte.

## Fazit für die Praxis

- Im Nachgang der Pandemie werden psychische Erkrankungen möglicherweise zunehmen. Um dem umfänglich Rechnung tragen und einer Überlastung bestehender Versorgungssysteme vorbeugen zu können, wird die Onlinetherapie (OT) in bestimmten Bereichen weiter eine wichtige Rolle spielen.
- Hierfür ist es notwendig, Aspekte wie Datenschutz, technische Rahmenbedingungen, Konnektivität, Bild‑, Tonqualität, Machbarkeit und Wirksamkeit weiter abzuklären.
- Die vorliegende Untersuchung konnte zeigen, dass therapeutenseitig eine prinzipielle Zugewandtheit und Zufriedenheit in Bezug auf OT vorhanden ist. Nichtsdestotrotz ist die Zufriedenheit mit der Face-to-face-Therapie höher. Weiterführende Untersuchungen, die die Gründe im Detail analysieren, sind sinnvoll.
